# Microbial genomic island discovery, visualization and analysis

**DOI:** 10.1093/bib/bby042

**Published:** 2018-06-03

**Authors:** Claire Bertelli, Keith E Tilley, Fiona S L Brinkman

**Affiliations:** Department of Molecular Biology and Biochemistry, Simon Fraser University, Burnaby, BC, Canada

**Keywords:** genomic island, horizontal gene transfer, microbial genomics, interactive visualization, antimicrobial resistance

## Abstract

Horizontal gene transfer (also called lateral gene transfer) is a major mechanism for microbial genome evolution, enabling rapid adaptation and survival in specific niches. Genomic islands (GIs), commonly defined as clusters of bacterial or archaeal genes of probable horizontal origin, are of particular medical, environmental and/or industrial interest, as they disproportionately encode virulence factors and some antimicrobial resistance genes and may harbor entire metabolic pathways that confer a specific adaptation (solvent resistance, symbiosis properties, etc). As large-scale analyses of microbial genomes increases, such as for genomic epidemiology investigations of infectious disease outbreaks in public health, there is increased appreciation of the need to accurately predict and track GIs. Over the past decade, numerous computational tools have been developed to tackle the challenges inherent in accurate GI prediction. We review here the main types of GI prediction methods and discuss their advantages and limitations for a routine analysis of microbial genomes in this era of rapid whole-genome sequencing. An assessment is provided of 20 GI prediction software methods that use sequence-composition bias to identify the GIs, using a reference GI data set from 104 genomes obtained using an independent comparative genomics approach. Finally, we present guidelines to assist researchers in effectively identifying these key genomic regions.

## A tale of genomic islands in bacterial evolution

Bacteria and archaea reproduce clonally, typically by binary fission/bipartition, but are also able to acquire foreign genetic material from other living organisms via horizontal gene transfer (HGT; also called lateral gene transfer). HGT has played an extensive role in microbial genome evolution, leading researchers to tentatively represent their evolution as a ‘Web of Life’ [1] or a ‘Rhizome of life’ [2] rather than a tree reflecting vertical descent. HGT is a major source of novel microbial genes, providing and maintaining diversity at the population level [3, 4]. Clusters of consecutive genes likely acquired by HGT are commonly described as genomic islands (GIs).

Several types of mobile genetic elements fall within this broad definition of GIs, including integrons, transposons, integrative and conjugative elements (ICEs) and prophages (integrated phages) [5]. These can be distinguished based on the mechanism of GI acquisition, mainly conjugation, transformation or transduction, and associated mobile selfish elements (integrases, transposases and insertion sequences) that promote GI mobilization and transfer [1, 6]. Once integrated into the genome, GIs evolve through mutations, genome rearrangements, gene loss or further acquisition of mobile genetic elements. The ability for a GI to transfer further to other microbial hosts strongly depends on the type of mobile genetic element, the host background and tightly regulated stochastic processes [6, 7].

Two groups of GIs can be distinguished with different roles: replacement and additive GIs [3]. Replacement GIs acquired by homologous recombination maintain the genomic diversity in the population and can be found conserved in distant relatives as well as in closely related strains [8]. Additive GIs are acquired by non-homologous recombination in preferential insertion sites such as transfer RNAs (tRNAs) or transfer-messenger RNAs (tmRNAs) or in the vicinity of highly conserved core genes, commonly leaving behind 16–20-bp direct repeats [9]. Additive GIs enable the bacterium to integrate multiple cassettes of different origin in a single genomic region, leading to their mosaic nature. The resulting genome flexibility enables rapid transfer of useful phenotypes in bacterial populations sharing a niche [10].

Genes that provide an advantage in a selective environment have been found to be associated with GIs, including those of significant medical interest and ‘novel’ genes (the latter reflecting a large, undersampled gene pool likely associated with these genomic regions) [11]. The spread of antimicrobial resistance (AMR) in some cases is the result of such HGT via bacterial conjugation [12] or phage transduction [13]. Virulence factors were also shown to be significantly disproportionately associated with GIs [14]. In particular, prophages can carry virulence factors that are often associated with increased bacterial virulence [15]. A recent study modeled how prophages, through recombination with actively infecting phages, play a key role in maintaining the phage population and expanding phage types [16]. They promote a diverse phage–host ecosystem with its associated large pool of genes driving microbial evolution. Indeed, lysogenic phages represent a major source of GIs [17, 18], as about 50% of characterized bacteria harbor one to over a dozen prophages [19].

The gene content of GIs was traditionally used to classify GIs into several subtypes, for example (i) pathogenicity islands (PAIs) that encode genes important for bacterial pathogenicity/virulence [20]; (ii) resistance islands that encode AMR genes [21]; (iii) symbiosis islands, as first coined for strains of *Mesorhizobium meliloti*, that are able to form nodules on plants after the acquisition of a 500-kb element [22];or (iv) metabolic islands that encode for adaptive metabolic abilities [6], for example the degradation of aromatic compound in *Pseudomonas knackmussii* [23]. However, the modular evolution of GIs and their composite nature enable single GIs to encode proteins with multiple functions. Hence, this simplistic classification reflects functional types driven by human interests for key gene functions rather than defined categories of evolutionary and mechanistic relevance.

As a direct consequence of their origin and evolution, features of GIs can include (i) a local nucleotide composition bias (guanine cytosine (GC) content, GC skew, codon usage, or k-mer signature), differing from the chromosome average; (ii) the presence of mobility genes and insertion sequences that can rapidly decay after GI integration; (iii) a high prevalence of phage-related genes; (iv) a high prevalence of hypothetical proteins; and (v) the presence of direct repeats [24].

## Computational prediction of GIs

GI detection, capitalizing on the features mentioned earlier, can be roughly classified into (1) sequence composition-based approaches and (2) comparative genomics approaches, based on the two most distinctive features associated with the horizontal origin of GIs: their sequence composition bias and sporadic phylogenetic distribution [5]. Despite the presence of well-known features, GI prediction is challenged by their mosaic nature and propensity to evolve rapidly through further gene acquisition that mixes nucleotide bias signatures, gene loss that can remove mobility genes or genome rearrangements. GI prediction has become an increasingly important component of bacterial genome investigation, and the development of new, more accurate tools has attracted major attention in the community, given the release of several new computational methods for GI prediction in the past decade (Table 1). Previous reviews of GI prediction methods have provided a good overall classification of tools available at the time of publication [5, 25, 26]. Hence, we review here new computational methods and new releases of existing software, in comparison with older methods.

**Table 1.** GI prediction tools listed by descending year of last publication

Predictors	Command line, GUI, webserver, database	Complete/draft genome	Input file	Year, reference	Link
IslandPath-DIMOB	C (W D)	C	gbk or embl	2018, [39]	https://github.com/brinkmanlab/islandpath
				2005, [11]	
IslandViewer	C W D	C / D	gbk or embl	2017, [47]	http://www.pathogenomics.sfu.ca/islandviewer
				2009, [67]	
Vrprofile	W, D	C / D	gbk or fna	2017, [55]	http://bioinfo-mml.sjtu.edu.cn/VRprofile/
MTGIpick	G W	C / D	fna	2016, [27]	http://bioinfo.zstu.edu.cn/MTGI/software.html
Zisland Explorer	C G W D	C	fna (optional ptt)	2016, [37]	http://tubic.tju.edu.cn/Zisland_Explorer/
MSGIP	C G	C	fna	2016, [34]	https://github.com/msgip/msgip
Islander	C D	C	fna	2015, [44]	http://bioinformatics.sandia.gov/islander/
				2004, [78]	
GI-SVM	C	C	fna	2015, [28]	https://github.com/icelu/GI_Prediction
PAI Finder-PAIDB^a^	W D	C	ffn (specific format)	2015, [58]	http://www.paidb.re.kr/
PIPS and GIPSy^b^	C G W	C	gbk or embl	2015, [54]	http://www.bioinformatics.org/groups/? group_id=1180
				2012, [53]	http://www.genoma.ufpa.br/lgcm/pips/
GIHunter	C D	C	fna, ptt, and rnt	2014, [43]	http://www5.esu.edu/cpsc/bioinfo/software/GIHunter/ http://www5.esu.edu/cpsc/bioinfo/dgi
Sighunt	C	C	fna	2014, [29]	https://www.iba.muni.cz/index-en.php? pg=research–data-analysis-tools–sighunt
GC-profile	C W	C	fna	2014, [36]	http://tubic.tju.edu.cn/GC-Profile/ and http://www.zcurve.net/
SVM-AGP (HGT)^a^	C		embl	2014, [79]	http://svm-agp.bioinf.mpi-inf.mpg.de/
GI-POP^a^	W	C / D	–	2013, [64]	http://gipop.life.nthu.edu.tw - deprecated
CGS (HGT)^a^	C	C	–	2012, [80]	available on request
EGID and GIST^a^	C G	C	fna, faa, ffn, gbk, and ptt	2012, [52]	http://www5.esu.edu/cpsc/bioinfo/software/GIST
				2011, [51]	http://www5.esu.edu/cpsc/bioinfo/software/EGID
IGIPT^a^	C W	C	fna/ffn	2011, [81]	http://bioinf.iiit.ac.in/IGIPT/
GIDetector^a^	G (C)	C	fna, ptt, rnt	2010, [42]	http://www5.esu.edu/cpsc/bioinfo/software/GIDetector/index.html
INDeGenIUS	C	C	fna	2010, [31]	available on request
MJSD	C	C	fna	2009, [35]	http://cbio.mskcc.org/∼aarvey/mjsd/
Design-Island^a^	G	C	fna	2008, [33]	http://www.isical.ac.in/∼rchatterjee/Design-Island.htm
PredictBias	W	C	gbk	2008, [66]	http://www.bioinformatics.org/sachbinfo/predictbias.html
RVM^a^	–	C	–	2008, [41]	no implementation available
IslandPick^b^	W D	C	gbk or embl	2008, [38]	http://www.pathogenomics.sfu.ca/islandviewer
DarkHorse^b^ (HGT)	C D	C / D	ffn	2007, [50]	http://darkhorse.ucsd.edu/
tRNAcc and MobilomeFinder^b^	C W D	C	fna, tRNA, ptt	2007, [49]	http://db-mml.sjtu.edu.cn/MobilomeFINDER/
				2006, [48]	
Centroid	C	C	ffn and ptt	2007, [30]	available on request
Colombo^a^	C G	C	embl, gbk, fasta	2006, [40]	http://www.uni-goettingen.de/en/research/185810.html
SIGI-CRF (HGT)	C (G)	C	embl	2006, [40]	http://www.uni-goettingen.de/en/research/185810.html
SIGI-HMM (HGT)	C (G)	C	embl	2006, [40]	http://www.uni-goettingen.de/en/research/185810.html
AlienHunter	C	C	fna	2006, [32]	http://www.sanger.ac.uk/science/tools/alien-hunter
Wn-SVM (HGT)	C	C	ffn and ptt	2005, [62]	available on request
PAI-IDA	C	C	gbk	2003, [63]	available on request

a Could not be successfully used for the GI predictor assessment analysis (Supplementary Table S1).

b Comparative genomics method and so excluded from the GI predictor assessment analysis.

() are used when the GUI, webserver or database has been published as a separate too.

### Sequence composition-based approaches

Assuming that mutational and selection pressures acting relatively homogeneously on microbial genomes create genome-wide signatures of nucleotide composition specific to each microbial species, genomic regions acquired by HGT can be distinguished from the rest of the genome in some cases by their atypical nucleotide composition. Methods adapted to the analysis of single genome sequences are generally based on the detection of such biases in sequence composition, eventually coupled with the analysis of gene content and further characteristics that are detailed in Table 2. Available tools usually examine k-mer frequencies or GC content at the genome or the gene level either using sliding windows or windowless methods. The wide variety of implementations, scoring algorithms and refinement methods (Tables 1 and 2) complicates the division of existing methods in intricate categories. Because of the large number of available methods, we will summarize here their similarities, advantages and drawbacks rather than precisely describing algorithms, as offered in previous reviews [5, 25].

**Table 2.** GI features used by GI predictors

Predictors	Method	Sequence composition bias	Machine learning	Other	Insertion site/ direct repeats	Mobility gene	Phage genes	Other genes
IslandPath-DIMOB	Seq composition	Dinucleotide	–	–	–	Integrase, transposase recombinase, insertion sequences	Yes	–
IslandViewer	Hybrid	Dinucleotide, codon usage	–	Incorporates Islander in pre-computed analyzes, and so contains its features	Incorporates Islander in pre-computed analyzes, and so contains its features	Integrase, transposase, recombinase, insertion sequences	Yes	VFs, AMRs
VRprofile	Hybrid	GC, dinucleotide, codon usage	–	BLASTP	tRNA, tmRNA, repeats	MobilomeDB	Yes	VFs, AMRs, secretion systems
MTGIpick	Seq composition	Tetranucleotide	–	–	–	–	–	–
Zisland Explorer	Seq composition	GC, codon usage, amino acid	–	–	–	–	–	–
MSGIP	Seq composition	Oligonucleotide	–	–	–	–	–	–
Islander	Structure	–	–	Negative filter: GC, length, integrase to end distance	tRNA, tmRNA, repeats	Integrase	–	–
GI-SVM	Seq composition	k-mer	SVM	–	–	–	–	–
PAI Finder-PAIDB^a^	Seq composition	GC, codon usage	–	–	–	–	–	VFs, AMRs
PIPS and GIPSy^b^	Hybrid	GC, codon usage	–	BLASTP	–	Transposase, tyr/ser recombinase	–	VFs, AMRs, metabolism, symbiosis, hypothetical proteins
GIHunter	Seq composition	k-mers, IVOM	Decision tree	Gene density, intergenic distance	tRNA	Integrase, transposase	Yes	–
Sighunt	Seq composition	Tetranucleotides	–	–	–	–	–	–
GC-profile	Seq composition	GC	–	–	–	–	–	–
SVM-AGP (HGT)^a^	Seq composition	GC, oligonucleotide, codon usage, amino acid, position-based frequency	SVM	–	–	–	–	–
GI-POP^a^	Seq composition	GC, oligonucleotide, codon usage, codon adaptation index	SVM	–	tRNA, repeats	Mobile genetic element from ACLAME database	–	–
CGS (HGT)^a^	Seq composition	k-mers	–	–	–	–	–	–
EGID and GIST^a^	Seq composition	Dinucleotide, codon usage, k-mers, IVOM	–	–	–	Integrase, transposase recombinase	–	–
IGIPT^a^	Seq composition	k-mers (2–6), codon usage, amino acid	–	–	–	–	–	–
GIDetector^a^	Seq composition	k-mers, IVOM	Decision tree	Gene density, intergenic distance	tRNA	Integrase, transposase	Yes	–
INDeGenIUS	Seq composition	k-mers	–	–	–	–	–	–
MJSD	Seq composition	k-mers	–	–	–	–	–	–
DarkHorse^b^ (HGT)	Comparative			BLAST, phylogeny				–
Design-Island^a^	Seq composition	GC, oligonucleotide	–	–	–	–	–	–
PredictBias	Seq composition	GC, dinucleotides, codon usage	–	–	tRNA	Insertion sequences	–	VFs
RVM^a^	Seq composition	k-mers, IVOM	RVM	Region size, gene density, insertion point	ncRNA, repeats	Integrase	Yes	–
IslandPick^b^	Comparative	–	–	Multiple-sequence alignment	–	–	–	–
tRNAcc and MobilomeFinder^b^	Comparative	–	–	Multiple-sequence alignment	tRNA	–	–	–
Centroid	Seq composition	k-mers	–	–	–	–	–	–
Colombo^a^	Seq composition	Codon usage, tetranucleotides	–	–	–	–	–	–
SIGI-CRF (HGT)	Seq composition	Tetranucleotides	–	–	–	–	–	–
SIGI-HMM (HGT)	Seq composition	Codon usage	–	–	–	–	–	–
AlienHunter	Seq composition	k-mers, IVOM	–	–	–	–	–	–
Wn-SVM (HGT)	Seq composition	k-mers	SVM	–	–	–	–	–
PAI-IDA	Seq composition	GC, dinucleotide, codon usage	–	–	–	–	–	–

a Could not be successfully used for the GI predictor assessment analysis (Supplementary Table S1).

b Comparative genomics method, and so excluded from the GI predictor assessment analysis.

#### Window-based and windowless methods analyzing genome-level nucleotide sequence composition

Many methods use sliding windows at the genome level (rather than analyzing individual genes) to calculate nucleotide composition biases, including MTGIpick [27], GI-SVM [28], SigHunt [29], Centroid [30], INDeGenIUS [31], AlienHunter [32] and Design-Island [33]. As reviewed in [25], these methods differ in the size of the sliding window, the scoring scheme as well as rules for the determination of regions with atypical composition. AlienHunter and GI-SVM use overlapping windows of fixed size, whereas MTGIpick, INDeGenIUS and Centroid use non-overlapping windows. Recently published, MTGIpick implements a *t*-test with selection of features to quantify compositional differences of tetranucleotides using kurtosis. It was suggested that it provides much higher recall, but lower precision, versus other window-based methods [27].

MSGIP [34], MJSD [35], GC-profile [36] and ZislandExplorer [37] are windowless methods using top-down approaches to determine break points in the genome sequence composition bias. GC-profile calculates GC content distribution, leaving the determination of GIs to the user. Its further development, named ZislandExplorer, refines regions identified using GC-profile by analyzing the codon and amino acid usage to define potential GIs. A recursive segmentation method is used by MJSD to identify changes in genome sequence atypicality, based on measures of Markov Jensen–Shannon divergence.

Most of these methods are sensitive but have low precision because of the limited information used for prediction (also see the benchmarking of GI predictors later). It is interesting to note that by further taking into account codon and amino acid usage, ZislandExplorer achieves a much improved precision compared with GC-profile. Overall, windowless methods supposedly better identify GI boundaries, as they do not rely on any predefined window size but can in principle determine boundaries as precisely as a single base. For window-based methods, the boundaries will depend on the size of the window used for computations. However, most methods do not provide specific implementations to improve the determination of GI boundaries, except MTGIpick that includes a derived version of MJSD to refine GI boundaries after an initial prediction step.

#### Window-based and windowless methods at the gene level

Genes represent an important functional unit of microbial genomes, which differ in sequence composition from non-coding regions of the genome, and were, therefore, used to identify composition biases or to refine predictions in many other methods (Table 2), including IslandPath-DIMOB, SIGI-HMM and SIGI-CRF, Wn-SVM, SVM-AGP, PredictBias, GIHunter, GIDetector and RVM. IslandPath-DIMOB and PredictBias both use a sliding window of six genes to calculate sequence composition bias, which essentially smooths the signal and, for example, avoids the detection of abnormal compositions because of high gene expression levels of small sets of genes. The use of criteria such as the presence of mobility genes in addition to sequence composition bias was shown to significantly improve the prediction accuracy of IslandPath-DIMOB by eliminating many false positives (FPs) [38].

A recently released version of IslandPath-DIMOB, with improved sensitivity in the score for nucleotide composition bias as well as novel HMM profiles to detect mobility genes, showed a highly increased recall with little loss of precision [39]. SIGI-HMM and SIGI-CRF, available as part of the Colombo package [40], measure codon usage bias in each gene as a signature for HGT. Individual genes that are predicted as having unusual codon usage using a Hidden Markov Model form potential GIs when closely positioned on the genome.

Following an assessment of structural features associated with GIs by Vernikos et al [41], machine learning approaches were developed, making use of the following GI features to train the models: insertion point, size of the region, gene density, presence of repeats, phage proteins, integrases and tRNAs (Table 2). GIDetector [42], a stand-alone software for Windows, and GIHunter [43], a Linux-compatible command line equivalent, have shown promising results in their ability to accurately predict GIs. Machine learning methods are able to integrate multiple GI features, improving the accuracy of GI predictions overall, as is observed for GIHunter [43].

#### Other sequence composition-based approaches

Finally, Islander [44] does not fall within any of the aforementioned categories, as it uses the presence of tRNA sequences and a ‘displacement fragment’ to predict the position of GIs. Further information, including a measure of GC content, and absence of a tyrosine integrase gene are used to filter out false positives (FPs). This method therefore only predicts a subset of GIs, though it predicts GI boundaries more effectively for those it does predict [44].

### Comparative genomics approaches

Comparative genomics approaches predict GIs based on the sporadic distribution of genomic regions acquired by HGT in closely related bacterial and archaeal genomes. Conversely, genomic regions conserved in large groups of monophyletic organisms are less likely to have horizontal origins. Comparative genomic methods have the advantage of determining precise GI boundaries based on multiple-sequence alignments or local alignments. However, they are dependent on the availability of appropriate closely related reference genomes used for comparison, and their prediction may vary according to the set of comparative reference genomes selected. Furthermore, they may be sensitive to prediction of genomic regions because of gene loss as well as HGT.

IslandPick [38] is the only method that performs an automatic selection of suitable genomes for comparison. Genomes are aligned using Mauve [45], and a secondary filter using BLAST [46] ensures that the predicted GI region is not simply a recent duplication or genomic rearrangement not aligned by Mauve. IslandPick predictions were shown to have a higher similarity to published GIs from the literature than other sequence composition-based methods [38], thus demonstrating the high accuracy of this comparative genomics approach. IslandPick is now available within the IslandViewer 4 webserver, which integrates multiple methods and provides a database of reference genomes for comparison [47]. IslandPick can also be customized, allowing a user to manually select the genomes to use for a comparative genomics-based prediction.

tRNAcc [48] and its webserver MobilomeFinder [49] use Mauve [45] multiple-sequence alignment to identify strain-specific regions downstream of tRNA genes that are predicted as GIs. The method is robust because of the combined requirement of a site-specific integration in a tRNA gene and the comparative genomic approach. However, it only identifies a subset of rather canonical GIs and is not able to predict GIs integrated in other genomic locations.

DarkHorse [50] has been designed to identify proteins likely acquired by HGT rather than large GIs. Protein sequences are subjected to BLAST analysis, comparing the sequences against the NCBI non-redundant database, and assigning a score reflecting atypical phylogenetic distance between the BLAST query and subject organisms. The combined approach using comparative genomics and phylogenetics identifies potential HGT candidates at different taxonomic levels. The use of amino acid sequences also enables analysis of more distantly related organisms, compared with IslandPick or tRNAcc.

### Hybrid and composite approaches

The various comparative genomics and sequence composition-based approaches, with their inherent advantages and drawbacks, have the potential to identify different regions acquired by HGT. Therefore, a few methods have implemented composite or hybrid approaches to improve GI prediction in bacterial and archaeal genomes (Table 2). The performance of such methods is tightly linked to the choice of individual tools combined as well as further decision rules for the integration of predictions.

EGID [51] and its graphical interface GIST [52] combine five sequence composition-based approaches: AlienHunter, COLOMBO SIGI-HMM, INDeGenIUS, IslandPath and PAI-IDA. To ponder the weight of each method, individual results are filtered to form non-overlapping regions in which gene scores exceed a threshold. These are further used to generate a consensus GI prediction by merging closely positioned regions. This composite approach was shown to achieve a balanced recall and precision of 0.63 when assessed using a reference data set identified by comparative genomics [51].

IslandViewer 4 integrates three composition-based methods (IslandPath-DIMOB, SIGI-HMM and Islander) as well as the comparative genomics method IslandPick. Related strains for genome comparison are automatically selected among the reference organisms available in the database, but users can also rerun the analysis using a different set of manually picked genomes. A database of precomputed genome analyses is also provided. However, there are some limitations for user-submitted genome analysis, versus precomputed genome analysis: IslandPick predictions are at the time of this publication restricted to the analysis of complete genomes, as the impact of poorly reordered contigs from draft genomes on its performance has not yet been assessed. Also, Islander predictions are so far only available for a subset of precomputed genomes, and not for custom user-supplied genome analyses. Overlapping predictions of the integrated tools in IslandViewer are merged, yielding a significant increase in recall (0.73) while maintaining good precision (0.9), thanks to limiting the methods chosen to be within IslandViewer as only those with relatively high precision [5, see also the analysis below].

PIPS [53] and its graphical user interface (GUI) GIPSy [54] determine the genomic signature deviation using custom scripts and SIGI-HMM, the presence of transposase genes and flanking tRNA genes and the absence in other organisms of the same genus or closely related species. A score assigned to each region, combining the presence of these characteristics, results in predictions at four levels of confidence. GIs are further classified into four subtypes based on the presence of factors for virulence, metabolism, antibiotic resistance or symbiosis. This tool is unique in that it combines sequence composition-based GI predictions with information on the conservation of genomic regions in user-provided custom genomes. However, the manual process in the graphical interface is tedious and reference genomes for comparison cannot be determined automatically, hindering its use for larger-scale analyses, which are becoming increasingly common.

VRprofile [55] performs sequence homology searches to a database of mobile genetic elements MobilomeDB, including notably prophages, ICEs, integrons and insertion sequences, taking into account gene order and clusters. It also uses and merges SIGI-HMM and IslandPath-DIMOB v0.2 predictions. Homology-based gene cluster and composition-based predictions are integrated, giving priority to gene clusters if they are larger than predicted GIs, and removing regions smaller than 5 kb. tRNAs and direct repeats are also predicted to better identify insertion sites and GI boundaries.

### Databases of GIs

Several websites hosting webservers and source code of GI predictors offer the possibility to download precomputed predictions for microbial genomes (indicated by a D in Table 1). As detailed later, some resources have further developed databases of predicted or curated GIs in publicly available genome sequences. Updated in 2015, the Islander [44] website provides 3919 GIs identified in 2168 prokaryotic genomes. Detailed information is available for each GI, including the position and sequence of tRNA integration site, the displaced tRNA fragment associated with the integration, biases in nucleotide composition and the gene content highlighting the integrase. Because it focuses on integrases of the tyrosine recombinase family, the Islander database also serves as a resource for the study of integrase site specificity and its evolution.

IslandViewer 4 [47] offers a database of precomputed GI predictions using three methods (IslandPath-DIMOB, SIGI-HMM and IslandPick) for 6193 bacterial isolates as well as Islander predictions for a subset of these isolates. These can be browsed in its interactive visualization interface, further highlighting the presence of AMR genes identified using the Comprehensive Antibiotic Resistance Database (CARD) [56], virulence factors (curated data set) as well as pathogen-associated genes [57]. Results can be downloaded in various text and graphical formats. The database is updated roughly semiannually, to include new genome sequences publicly available in RefSeq.

VRprofile [55] offers precomputed predictions for 2428 complete genomes. However, we were only able to download the list of ‘candidate genes’, including mobility genes, AMR genes and virulence factors, as well as insertion sequences.

The Pathogenicity Island Database PAIDBv2.0 [58] contains 1331 PAIs and 108 AMR islands from 2673 prokaryotic genomes collected from the literature. Candidate regions identified using SIGI-HMM [40] and IslandPath-DIMOB v0.2 [11] are also reported. For each GI, a graphical and tabular representation of the gene content, tRNA and insertion sequences/direct repeats provides direct links to the sequence. The database also lists publications associated with the discovery of a GI, as well as other genomes exhibiting sequence similarity to the GI identified.

ICEberg [59] is a database of ICEs in 363 bacterial isolates with experimental data extracted from the literature (*n* = 211), predicted by bioinformatics methods (*n* = 219) or directly extracted from GenBank (*n* = 30). An organism or ICE family-based browser displays detailed information on 460 ICEs, including a genome context view, sequence information as well as related publications.

Finally, the DarkHorse database [60] identifies phylogenetically atypical proteins probably acquired by HGT with various degrees of phylogenetic granularity (strain, species and genus) in 1456 bacterial and archaeal genomes (last updated in 2009). Results are returned in a tabular format with links to the protein sequence.

## Benchmark of GI predictors

To enable researchers to select the optimal tool for their needs, it is essential to have large-scale assessments and comparisons of available methods. In 2008, a first assessment of GI predictors by Langille *et al.* [38] using a data set of GIs identified by the comparative method IslandPick represented a landmark in the field, as it enabled researchers to compare the performance of several popular methods for GI prediction. Since then, numerous methods to predict GIs have been published, claiming significant improvements in recall and accuracy compared with existing methods. However, different positive reference data sets of GIs are used in each publication to demonstrate the predictor accuracy, precluding a direct comparison of the methods. A major limitation of most articles is that the reference data set is often limited to one or only a few genomes to provide a proof of concept for the new method, which does not represent the breadth/variety of microbial genomes. To provide guidance for researchers who need to analyze one to hundreds of bacterial genomes, we have performed an assessment of GI prediction tools, using one comparative data set of islands from 104 genomes, as well as a literature data set of six genomes, plus a negative data set [39].

Among the 37 methods identified for this review (Table 1), including command line tools, GUI-based tools and webservers, we could not find the web resources or source code for only two tools. Eight tools could not be run because of various reasons (Supplementary Table S1), mostly software errors, some of which might be linked to our operating systems but given the number of tools considered here and time constraints could not all be individually solved. The six tools that include comparative genomics approaches (Supplementary Table S1) have been excluded from this analysis because their results largely depend on the genomes chosen as a reference and most tools do not provide an automatic selection of phylogenetically closely related genomes for the analysis. Altogether, 20 composition-based prediction tools were successfully used to predict GIs on a data set of 104 bacterial genomes described in [39].

### Methods

The reference data sets described in [39] comprise a positive data set of GIs predicted by comparative genomics using IslandPick combined from Langille *et al* [38], IslandViewer 3 [61] and IslandViewer 4 [47] as well as a negative data set of core conserved genomic regions [38]. It includes 104 genomes, representing 22 different bacterial genera and 53 species that cover several common pathogens and environmental bacteria. Each genome encodes between 1 and 77 predicted GIs (mean = 17.7, SD = 16.6), for a total of 1845 GIs. The full data set is available for download in tabular and fasta format at the following URL: http://www.pathogenomics.sfu.ca/islandviewer/download/. The literature positive data set includes curated GIs recovered in the literature for six bacterial genomes: *Escherichia coli* O157: H7 str. Sakai (NC_002695.1)*, Escherichia coli CFT073* (NC_004431.1), *Salmonella enterica subsp. enterica serovar Typhi* str. CT18 (NC_003198.1), *Staphylococcus aureus* str. MW2 (NC_003923.1), *Streptococcus pyogenes* str. MGAS315 (NC_004070.1) and *Vibrio parahaemolyticus* RIMD 2210633 (NC_004603.1). Recall, precision, accuracy, *F*-score and Matthews correlation coefficient (MCC) were calculated as in [39], based on the number of true positives (TPs) and (FPs) measured as the number of nucleotides in predicted GIs overlapping with the positive and negative data sets, respectively, and true negatives (TNs) and false negatives (FNs) measured as the number of nucleotides outside predicted GIs overlapping with the negative and positive data sets, respectively:

$$Recall=TPR=\frac{TP}{TP+FN}$$

$$Precision=PPV=\frac{TP}{TP+FP}$$

$$Overall\;accuracy=ACC=\frac{TP+TN}{TP+FP+FN+TN}$$

$$F1\;score=F1=\frac{2TP}{2TP+FP+FN}$$

$$MCC=\frac{TPxTN-FPxFN}{\sqrt{\left(TP+FP)(TP+FN)(TN+FP)(TN+FN\right)}}$$

When TP and FP were equal to 0, the precision was counted as equal to 1 because the software is being conservative making no prediction. The MCC varies between -1 (complete disagreement between the prediction and the reference data set) and 1 (perfect prediction). An MCC of 0 indicates a prediction no better than random. MCC was considered as 0 when the denominator was equal to 0. The code used to assess GI predictors is available at https://github.com/brinkmanlab/gi_predictor_assessment.

All stand-alone software was installed on Debian 3.2.41 x86_64 machines, CentOS 7 machines for GI-SVM and GCProfile and Windows 10 for GIDetector and Design-Island. Webservers were accessed using the URLs in Table 1. Default parameter values were used for all software, as built into the program, as described in the readme document or in the corresponding publication. In the case of MJSD, a significance threshold of 0.99, segmentation model order of 1 and atypicality assessment model order of 2 were used, as in the article. In the case of Wn-SVM [62], parameters -nu of 0.2 and -n of 6 to run the analysis were used, as suggested by Dr Tsirigos. MJSD [35], PAI-IDA [63], SigHunt [29], GC-Profile [36] and INDeGenIUS [31] returned a numerical score as a result and thus required a cutoff to distinguish GIs. For MJSD, *Salmonella enterica* strain NC_003198.1 was analyzed and compared with the results described in the article [35]. Classifying segments with scores equal or greater than 0.99999 as GIs was found to have the strongest resemblance. The cutoffs 3.9 and 5 were used for PAI-IDA and SigHunt, as suggested in their respected publications. No recommended cutoff was available for INDeGenIUS and GC-Profile, as these softwares produce scores dependent on the composition of the genome being analyzed. For INDeGenIUS, regions with scores exceeding 2 SDs from the mean were considered GIs. For GC-Profile, regions with scores beyond 2 weighted SDs from the weighted mean, in either direction, were classified as GIs. The weights were based on the length of the genomic regions considered.

### Results

**Figure 1.**
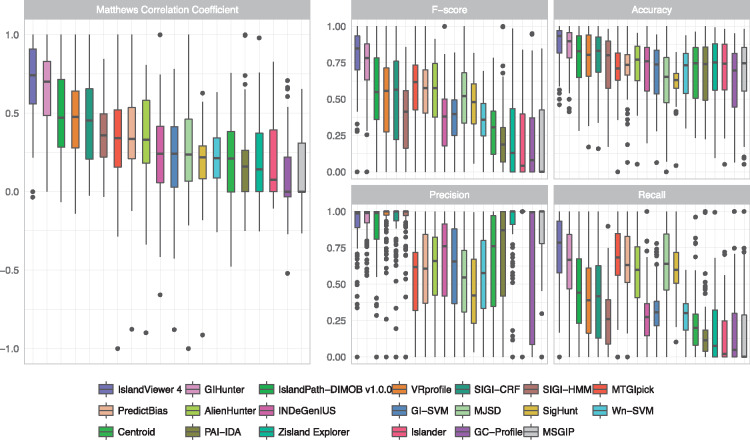
Accuracy assessment of genomic island prediction methods. Accuracy of genomic island (GI) predictors was assessed using a data set of GIs identified by comparative genomics analysis of 104 genomes [39]. Each genome is represented by a value, with the median, and the first and third quartiles represented in the boxplot as the lower and upper hinges, respectively. Outliers are shown as black dots, if they exceed 1.5 times the interquartile range.

GI predictors differ greatly in their ability to accurately predict GIs, with consistent trends among the 104 genomes used here in the reference comparative data set (Figure 1). All tools also show significant variation in their recall, precision and accuracy among the 104 genomes, stressing the need to use a diverse and large data set of genomes to assess GI predictors to avoid biases because of the selection of specific bacterial taxa on which some tools might perform better. Notably, average values for some tools (Table 3) can differ greatly from the median (Figure 1, black horizontal line in the boxes) because of the presence of a number of outliers.

**Table 3.** Mean GI prediction accuracy (%) assessed using the reference comparative genomics-based data set listed by descending MCC

Predictors	MCC	*F*-score	Accuracy	Precision	Recall
IslandViewer 4	0.703	0.780	0.880	0.904	0.732
GIHunter	0.645	0.713	0.849	0.934	0.634
IslandPath-DIMOB v1.0.0	0.486	0.554	0.768	0.874	0.469
VRprofile	0.470	0.511	0.774	0.940	0.417
SIGI-CRF	0.441	0.498	0.784	0.920	0.400
SIGI-HMM	0.353	0.374	0.729	0.919	0.264
PredictBias	0.347	0.526	0.715	0.577	0.623
AlienHunter	0.342	0.540	0.734	0.594	0.570
MTGIpick	0.324	0.559	0.704	0.551	0.675
MJSD	0.259	0.497	0.614	0.521	0.638
INDeGenIUS	0.236	0.356	0.699	0.651	0.275
GI-SVM	0.221	0.382	0.685	0.603	0.317
Wn-SVM	0.204	0.354	0.685	0.552	0.286
Zisland Explorer	0.203	0.239	0.688	0.853	0.177
Centroid	0.196	0.292	0.678	0.627	0.217
Islander	0.191	0.204	0.697	0.971	0.140
SigHunt	0.186	0.466	0.619	0.457	0.605
PAI-IDA	0.177	0.220	0.671	0.685	0.149
MSGIP	0.150	0.199	0.678	0.865	0.163
GC-Profile	0.091	0.205	0.620	0.637	0.225

On average, SIGI-HMM, INDeGenIUS, GI-SVM, Wn-SVM, Zisland Explorer, Centroid, Islander, PAI-IDA, GC Profile and MSGIP have rather low (<0.32) recall, whereas IslandPath-DIMOB, SIGI-CRF and VRprofile show intermediate recall (0.4–0.47), and IslandViewer 4, GIHunter, PredictBias, AlienHunter, MTGIpick, MJSD and SigHunt have higher recall (0.57–0.73). However, when choosing a predictor, researchers also need to consider the precision, which reflects the software tendency to make FP predictions. Some tools with very low recall to intermediate recall (IslandPath-DIMOB, SIGI-CRF, SIGI-HMM, ZislandExplorer, VRprofile and Islander) have high precision, with Islander at the extreme exhibiting a very low recall but a constantly high precision thanks to its strict criteria to predict a subset of GIs integrated in tRNAs or tmRNAs [44]. Several predictors (PredictBias, AlienHunter, MTGIpick, MJSD and SigHunt) with high recall exhibit a poor precision (0.46–0.59). Overall, sequence composition-based approaches working at the gene level generally present more robust predictions than those analyzing genome-level sequences (greater accuracy; *P* < 0.01), with lower recall but much higher precision than the aforementioned genome-based methods.

Only IslandViewer 4, a composite method that includes IslandPick comparative genomic approach, SIGI-HMM and IslandPath-DIMOB [47], as well as GIHunter, a single method that incorporates multiple features to build a decision tree for GI prediction [43], show both a high recall and precision. This is reflected in the high overall accuracy, *F*-score and especially MCC values obtained by those two tools compared with the others. MCC is widely used for software benchmarking and is considered a balanced measure of the correlation between the reference data sets and the observed predictions, robust to different sizes in the confusion table. However, it must be emphasized that for some users, high precision is critical, whereas for others, high recall is essential, and so, a collection of measures is shown.

The positive data set identified by comparative genomics comprises many genomes from different bacterial species and includes some well-known GIs identified in the literature [38, 39]. However, GIs in this comparative genomics data set have not been manually curated. GI predictors were thus also evaluated using a positive data set of GIs retrieved from the literature in six bacterial genomes (Table 4). This data set also allows an unbiased assessment of IslandViewer and avoids the circularity linked to the use of IslandPick to build the reference data set and its integration in IslandViewer 4 webserver predictions. The small number of genomes considered here and their biased representation reduces the statistical significance of the analysis, and results should be taken with caution. Nevertheless, note the general congruence of the results (Pearson’s correlation coefficient of 0.86). Several methods show highly improved MCC, such as PredictBias, VRprofile, MTGIpick, Islander and MSGIP, notably thanks to the much higher recall observed. The better performance of tools that require the presence of features of GIs, such as Islander, VRprofile or GIHunter, which were trained on such a literature data set, could partially reflect this training, and the tendency to describe large canonical GIs in the literature rather than smaller regions that are equally well identified by a comparative genomics approach such as IslandPick. As the negative data set and thus FPs remain the same as with the comparative data set, changes in precision are only because of the increase of TPs (also visible from the increased recall) and the choice of a small number of genomes in this literature data set. The creation of a larger data set of literature-based curated GIs, and corresponding negative data sets, would be key to better benchmark GI predictors and assess their ability to predict large multi-modular GIs. GIHunter and IslandViewer 4 remain among the methods with the highest MCC together with PredictBias, although more closely followed by other GI predictors using this literature data set for evaluation.

**Table 4.** Mean GI prediction accuracy (%), assessed using the reference literature-based data set and listed by descending MCC

Predictor	MCC	*F*-score	Accuracy	Precision	Recall
GIHunter	0.734	0.832	0.847	0.981	0.745
PredictBias	0.643	0.838	0.820	0.868	0.817
IslandViewer 4	0.640	0.753	0.788	0.998	0.619
VRprofile	0.574	0.643	0.751	0.993	0.542
IslandPath-DIMOB v1.0.0	0.541	0.669	0.720	0.979	0.521
MTGIpick	0.504	0.775	0.753	0.819	0.744
SIGI-CRF	0.426	0.522	0.689	0.993	0.436
AlienHunter	0.398	0.642	0.705	0.753	0.570
SIGI-HMM	0.359	0.420	0.600	0.998	0.285
MJSD	0.347	0.694	0.653	0.742	0.697
Islander	0.321	0.354	0.560	1.000	0.226
MSGIP	0.306	0.439	0.620	0.947	0.353
SigHunt	0.234	0.649	0.620	0.692	0.624
Zisland Explorer	0.175	0.264	0.520	0.833	0.171
INDeGenIUS	0.152	0.293	0.515	0.716	0.189
GI-SVM	0.150	0.312	0.512	0.721	0.200
Wn-SVM	0.129	0.320	0.499	0.715	0.208
PAI-IDA	0.077	0.118	0.461	0.667	0.067
Centroid	0.075	0.202	0.481	0.636	0.129
GC-Profile	−0.015	0.063	0.414	0.667	0.034

### Ease of use, common issues and solutions

Researchers interested in using some of these GI predictors may face difficulties accessing the software code and installing it, obtaining the correct input files and interpreting the results—in particular when requiring predictions for large numbers of isolates with minimal manual handling. Early GI predictors were developed as command line software in various coding languages, with a tendency to publish GUIs, webservers or databases providing an easier access for non-bioinformaticians in the following years (Table 1). GI predictors developed recently all include a GUI and/or a webserver as do some other older successful predictors and databases with good long-term maintenance such as Colombo [40], PAIDB [58] and IslandViewer [47]. To help researchers access existing and adapted tools for their needs, we have summarized key features of the software investigated in Table 1. More detailed information is available in Supplementary Table S1.

An important criterion for choosing a suitable GI predictor can be the type of input file required. Whereas, some tools require simple nucleotide fasta files (.fna and .ffn for genes), or standard flat file formats from nucleotide repositories such as GenBank (.gbk, .gbff and .gb) or Embl (.embl), others require tabular formats that are no longer provided by default in nucleotide repositories (.ptt and .rnt; Table 1). These latter tables can be inferred from standard flat file formats using custom scripts, but they therefore require some knowledge of a coding language.

Also, with the democratization and the popularity of short-read sequencing, most sequences currently submitted to public databases are draft/incomplete genomes (i.e. they include several contigs). Most tools only accept complete genomes as input, except (theoretically) five webservers. IslandViewer 4 is one such tool, which provides the possibility to select a reference genome for contig reordering before GI prediction [47]. According to its publication, MTGIpick accepts fasta files with multiple contigs [27], but the corresponding website indicates that only files with one nucleotide sequence are accepted. PAI-Finder accepts nucleotide fasta files of genes and can, therefore, handle unfinished genomes but with a limit of 1000 sequences per submission [58]. GI-POP, a webserver previously accepting draft genomes, has not been accessible since publication [64]. Finally, VRprofile website provides a tool called CDSeasy to annotate and reorder contigs according to a complete reference genome that provides a gbk file with a pseudochromosome that can be used as input to VRprofile [55]. To overcome limitations and use other GI predictors on draft genomes, a common strategy is to reorder contigs according to a closely related complete reference genome using tools such as Mauve [45] or ABACAS [65] and concatenate them in a single pseudochromosome. Contigs that could not be reordered are generally placed at the end of the pseudochromosome. Predictions on such pseudochromosomes should be interpreted with caution, considering the position of gaps between contigs and the possibility that some smaller contigs belonging to the GI could not be placed correctly. Indeed, transposases and integrases that are features of GIs and used by many prediction software are often found in single small contigs because such mobile elements exist in several identical copies in the genome, leading to difficulties in genome assembly. Finally, the quality of the draft genome (fewer contigs is better) and the genetic distance with the reference genome (the closest reference is generally better) may affect the reordering and the quality of GI predictions.

Several predictors have flexible input parameters to perform the analysis. Although this is desirable, to customize predictions for particular genomes of interest, this can become problematic when defaults are lacking or recommended parameters are not clearly stated. Similarly, some GI predictors such as INDeGenIUS, MJSD, PAI-IDA, SigHunt and GC-Profile return a numerical score or bias value that needs to be interpreted to infer the position of GIs along the genome. This flexibility often comes at the expense of software ease of use—particularly for larger-scale analyses. Indeed, in the absence of closely related reference genomes with highly curated GIs from literature that can be used as benchmark, optimal parameters and cutoffs may be difficult to choose, given the wide variation in prediction accuracy among different bacterial species. All parameters and cutoffs used in this analysis (defaults, suggested with the software or stated in the publication) are mentioned in Supplementary Table S1.

Finally, most current microbial genome projects now involve the sequencing of many isolates. These large-scale analyses require adapted tools to minimize manual handling time that is notably usually required for submissions to webservers. Command line tools, which comprise most methods currently available, are well adapted to batch submissions for researchers with some basic knowledge and coding ability. Other tools available as webservers only, such as PredictBias [66], can be automatized using packages such as Selenium Python, but this requires more advanced coding knowledge. The recently released IslandViewer 4 [47] offers an HTTP API for submission of many genomes and retrieval of results using various coding languages or simple cURL commands.

### Choosing a GI predictor

**Figure 2.**
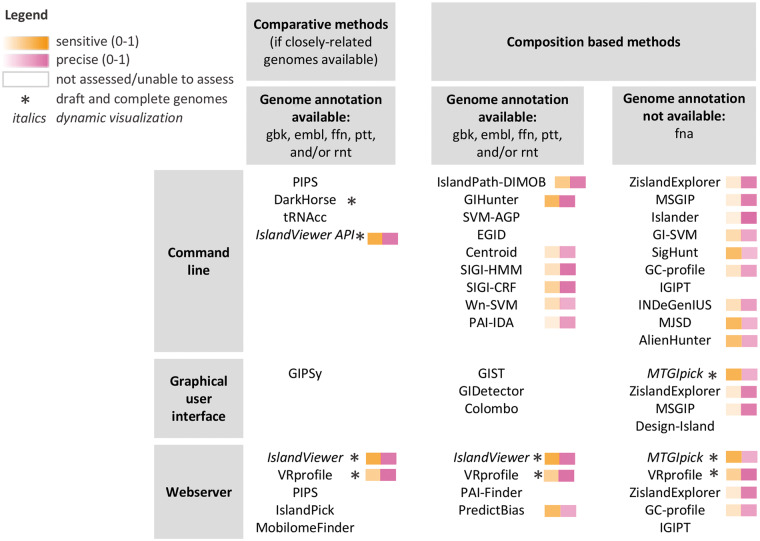
Summary of GI predictor characteristics with a multi-entry decision table. GI predictors were classified according to their interface and the requirements. Sensitivity (recall) and precision are color coded using a gradient from low-to-high as assessed using the comparative genomics data set. Methods that were not assessed (comparative genomics) or that we were unable to assess are shown in white.

Given the variety of GI predictors and their diverse features, the choice of a tool will depend on characteristics of the genome to be analyzed as well as on requirements of the planned research. The availability of closely related genomes is necessary to use methods based on comparative genomics that generally enable a more precise definition of GI boundaries. Adapted to single genomes, the numerous methods based on sequence composition are commonly used. Depending on the research focus, methods with lower recall but very high precision may be of interest, to ensure robust predictions, or conversely, high recall may be desired to reduce the chance of missing predicting a key GI region. For example, focusing on canonical GIs integrated in tRNAs would require software such as Islander [44] or tRNAcc [49] that use the presence of a tRNA integration site as a requirement to issue a GI prediction (Table 2); however, this method will miss the majority of islands, which are non-canonical. Depending on the tool, composition-based prediction methods may have less accurate determination of GI boundaries and will require careful inspection. In addition, the availability of genome annotation as well as the status of the genome assembly, complete or incomplete/draft including several contigs, largely reduce the choice of possible tools. To facilitate the selection of an appropriate tool, we have summarized the characteristics of all tools and provide a multi-entry decision table in Figure 2.

Because of the varying sensitivity and criteria used by GI predictors, predictions obtained using different tools generally only partially overlap. As previously observed [5] and used for the development of composite methods such as IslandViewer [67], VRprofile [55] and EGID [51], combining several methods results in a significant increase in the recall of GIs. In general, the selection of highly precise methods is advisable to preserve a good prediction accuracy, as shown earlier for IslandViewer 4 and VRprofile. Finally, the combination of both composition-based and comparative genomics methods has the potential to identify GIs with different characteristics.

## GI visualization and future needs

Although there is a notable trend toward providing GUIs and webservers to enable non-bioinformaticians to run GI predictions on user-provided genomes, most interfaces only have limited ability to visualize GIs. GIPSy [54], GC-profile [36] and PredictBias [66] only provide GIs as tabular results. PredictBias further displays a tabular list of genes encoded in the predicted GIs, as well as potential VFs. ZislandExplorer [37] provides static images with GIs highlighted, whereas Islander [44] and VRprofile [55] display static graphs of genomic features as well as tabular results. Che *et al.* have developed a C++ tool named GIV [68] to plot AlienHunter or GIHunter predictions and GI-associated features in a circular representation using Circos [69].

Only a few resources provide more advanced visualization techniques. According to the publication, MTGIpick [27] presents a dynamic GI visualization in the form of a circular representation of genomes with predicted GIs that allow the user to zoom in and out. However, we were unable to successfully run MTGIpick stand-alone version. PAIDB [58] displays linear views of precomputed GI predictions featuring genes as arrows on the two strands. Clicking on any arrow displays additional information, including gene function, sequences and related publications. IslandViewer provides by far the most extensive, integrated and interactive visualization of GIs and their genetic content, highlighting virulence factors (VFs), AMR determinants and pathogen-associated genes [47]. Gene content can be browsed in a pop-up table with direct links to public databases such as NCBI for publicly available genomes, as well as CARD [56] for AMR, and VFDB [70], Victor’s virulence (http://www.phidias.us/victors/) and PATRIC [71] for VFs. Since IslandViewer 3 release [61], synchronized circular and linear views with intuitive zoom and navigation features represent GIs broken down by a prediction method using GenomeD3Plot [72]. Moreover, IslandViewer allows the independent visualization of two genomes side by side, as a first step to enable GI comparison between bacterial isolates.

Further development of advanced visualization techniques is required to facilitate the analysis of the key role of GIs in bacterial adaptability. Indeed, integrated views with AMR genes and VFs are a critical step to investigate bacterial evolution, particularly in the context of infectious disease outbreaks, which are now becoming routinely investigated using whole-genome sequencing (termed genomic epidemiology) [73, 74]. For environmental strains, automatic identification of metabolic pathways or transporters that could enable the microbe to thrive on different food sources would be valuable. Further identification and highlighting of interesting features such as phage genes, mobility genes (integrases, transposases), preferential insertion sites such as tRNAs as well as bordering direct repeats (when existing) would facilitate the analysis of the genome dynamic and the origin of these horizontally acquired genomic regions. Finally, and perhaps most pressingly, there is a high need to visualize comparative genomic analyses at a larger scale. Specific interfaces must be developed to accommodate the need to smoothly and interactively visualize up to hundreds of microbial genomes and their GI predictions, identifying clusters of related islands and rearrangements of potential interest.

## Moving toward integrated analyses beyond the single genome

There has been a resurgence of interest in GIs in recent years, as large-scale microbial genome analyses, including genomic epidemiological analyses, further reveal the importance of GIs in microbial adaptation to environmental conditions—including medically relevant adaptations by pathogens. The prediction of GIs and the analysis of their gene content have become an essential component of microbial investigations, from genome reports to detailed comparative genomics studies. In particular, in clinical microbiology and epidemiology, it is increasingly needed to rapidly identify recently acquired genomic regions that may be unique to a disease outbreak, or associated with a pathogen strain that has a particular phenotype of interest, such as increased persistence, transmissibility, AMR or resistance to solvents used for their control, as investigated for Listeria *monocytogenes* [75], Pseudomonas *aeruginosa* [76] and *E. coli* [77]. Many genomes can now be sequenced daily and made available rapidly using a single short-read sequencer or certain longer-read technologies, such that sequence analysis represents the major bottleneck. Among the many tools currently available to predict GIs presented in this review, each method has its strengths and weaknesses depending on the type of genomic data available as well as research requirements, but a combination of methods is often most suitable and desirable. Predicted GIs should be manually checked and their gene content searched for genes of medical or environmental interest, boundaries eventually refined, and GIs can be further compared using homology searches. Further analysis of integration sites and GI conservation with further modular gene acquisition or loss across larger numbers of samples are desirable to obtain a better picture of bacterial genome evolution. Tools to predict, analyze and visualize GIs need to accommodate the need for better-integrated evaluation of microbial genomes. The development of more comprehensive methods to identify GIs, their origin and their encoded features will enable researchers to implement real-time tracking of microbial genome evolution in answer to changing environmental conditions and selection pressures such as antibiotic use.

Key Points:

- GIs are clusters of microbial genes that were likely horizontally acquired. They are key players in genome evolution, facilitating microbial adaptability of wide environmental, industrial and medical interest.
- GI predictors show highly varying levels of recall and precision with consistent trends across diverse microbial species.
- Benchmarking of new GI predictors should preferably be performed on large and varied data sets of bacterial/archaeal isolates.
- More integrated interactive analysis and visualization of GIs in multiple genomes is now required, as the demand for large-scale analyses increases.

## Supplementary Data

Supplementary data are available online at https://academic.oup.com/bib.

## Supplementary Material

bby042_Supp

## References

[1] Soucy SM, Huang J, Gogarten JP. Horizontal gene transfer: building the web of life. Nat Rev Genet 2015;16(8):472–82. 26184597 10.1038/nrg3962

[2] Raoult D. The post-Darwinist rhizome of life. Lancet 2010;375(9709):104–5. 20109873 10.1016/S0140-6736(09)61958-9

[3] Rodriguez-Valera F, Martin-Cuadrado A-B, López-Pérez M. Flexible genomic islands as drivers of genome evolution. Curr Opin Microbiol 2016;31:154–60. 27085300 10.1016/j.mib.2016.03.014

[4] Niehus R, Mitri S, Fletcher AG, et al Migration and horizontal gene transfer divide microbial genomes into multiple niches. Nat Commun 2015;6:8924. 26592443 10.1038/ncomms9924 PMC4673824

[5] Langille MGI, Hsiao WWL, Brinkman FSL. Detecting genomic islands using bioinformatics approaches. Nat Rev Microbiol 2010;8:373–82. 20395967 10.1038/nrmicro2350

[6] Juhas M, van der Meer JR, Gaillard M, et al Genomic islands: tools of bacterial horizontal gene transfer and evolution. FEMS Microbiol Rev 2009;33(2):376–93. 19178566 10.1111/j.1574-6976.2008.00136.x PMC2704930

[7] Minoia M, Gaillard M, Reinhard F, et al Stochasticity and bistability in horizontal transfer control of a genomic island in *Pseudomonas*. Proc Natl Acad Sci USA 2008;105:20792–7. 19098098 10.1073/pnas.0806164106 PMC2605633

[8] Choi SC, Rasmussen MD, Hubisz MJ, et al Replacing and additive horizontal gene transfer in streptococcus. Mol Biol Evol 2012;29(11):3309–20. 22617954 10.1093/molbev/mss138 PMC3472495

[9] Williams KP. Integration sites for genetic elements in prokaryotic tRNA and tmRNA genes: sublocation preference of integrase subfamilies. Nucleic Acids Res 2002;30:866–75. 11842097 10.1093/nar/30.4.866 PMC100330

[10] Jasni AS, Mullany P, Hussain H, et al Demonstration of conjugative transposon (Tn5397)-mediated horizontal gene transfer between *Clostridium difficile* and *Enterococcus faecalis*. Antimicrob Agents Chemother 2010;54:4924–6. 20713671 10.1128/AAC.00496-10 PMC2976158

[11] Hsiao WWL, Ung K, Aeschliman D, et al Evidence of a large novel gene pool associated with prokaryotic genomic islands. PLoS Genet 2005;1(5):e62. 16299586 10.1371/journal.pgen.0010062 PMC1285063

[12] von Wintersdorff CJH, Penders J, van Niekerk JM, et al Dissemination of antimicrobial resistance in microbial ecosystems through horizontal gene transfer. Front Microbiol 2016;7:173. 26925045 10.3389/fmicb.2016.00173 PMC4759269

[13] Brown-Jaque M, Calero-Cáceres W, Muniesa M. Transfer of antibiotic-resistance genes via phage-related mobile elements. Plasmid 2015;79:1–7. 25597519 10.1016/j.plasmid.2015.01.001

[14] Ho Sui SJ, Fedynak A, Hsiao WWL, et al The association of virulence factors with genomic islands. PLoS One 2009;4(12):e8094. 19956607 10.1371/journal.pone.0008094 PMC2779486

[15] Davies EV, Winstanley C, Fothergill JL, et al The role of temperate bacteriophages in bacterial infection. FEMS Microbiol Lett 2016;363(5):fnw015. 26825679 10.1093/femsle/fnw015

[16] Nadeem A, Wahl LM. Prophage as a genetic reservoir: Promoting diversity and driving innovation in the host community. Evolution 2017;71:2080–9. 28590013 10.1111/evo.13287

[17] Brüssow H, Canchaya C, Hardt W-D. Phages and the evolution of bacterial pathogens: from genomic rearrangements to lysogenic conversion. Microbiol Mol Biol Rev 2004;68:560–602. 15353570 10.1128/MMBR.68.3.560-602.2004 PMC515249

[18] Casjens S. Prophages and bacterial genomics: what have we learned so far? Mol Microbiol 2003;49(2):277–300. 12886937 10.1046/j.1365-2958.2003.03580.x

[19] Touchon M, Bernheim A, Rocha EP. Genetic and life-history traits associated with the distribution of prophages in bacteria. ISME J 2016;10(11):2744–54. 27015004 10.1038/ismej.2016.47 PMC5113838

[20] Hacker J, Bender L, Ott M, et al Deletions of chromosomal regions coding for fimbriae and hemolysins occur *in vitro* and *in vivo* in various extraintestinal *Escherichia coli* isolates. Microb Pathog 1990;8:213–25. 1974320 10.1016/0882-4010(90)90048-u

[21] Juhas M, Power PM, Harding RM, et al Sequence and functional analyses of *Haemophilus* spp. genomic islands. Genome Biol 2007;8(11):R237. 17996041 10.1186/gb-2007-8-11-r237 PMC2258188

[22] Sullivan JT, Ronson CW. Evolution of rhizobia by acquisition of a 500-kb symbiosis island that integrates into a phe-tRNA gene. Proc Natl Acad Sci USA 1998;95:5145–9. 9560243 10.1073/pnas.95.9.5145 PMC20228

[23] Miyazaki R, Bertelli C, Benaglio P, et al Comparative genome analysis of *Pseudomonas knackmussii* B13, the first bacterium known to degrade chloroaromatic compounds. Environ Microbiol 2015;17(1):91–104. 24803113 10.1111/1462-2920.12498

[24] Dobrindt U, Hochhut B, Hentschel U, et al Genomic islands in pathogenic and environmental microorganisms. Nat Rev Microbiol 2004;2:414–24. 15100694 10.1038/nrmicro884

[25] Lu B, Leong HW. Computational methods for predicting genomic islands in microbial genomes. Comput. Struct Biotechnol J 2016;14:200–6. 10.1016/j.csbj.2016.05.001 PMC4887561 27293536

[26] Che D, Hasan MS, Chen B. Identifying pathogenicity islands in bacterial pathogenomics using computational approaches. Pathogens 2014;3:36–56. 25437607 10.3390/pathogens3010036 PMC4235732

[27] Dai Q, Bao C, Hai Y, et al MTGIpick allows robust identification of genomic islands from a single genome. Brief Bioinform 2016;19:361–73. 10.1093/bib/bbw118 PMC6454522 28025178

[28] Lu B, Leong HW. GI-SVM: a sensitive method for predicting genomic islands based on unannotated sequence of a single genome. J Bioinform Comput Biol 2016;14(01):1640003. 26907990 10.1142/S0219720016400035

[29] Jaron KS, Moravec JC, Martínková N. SigHunt: horizontal gene transfer finder optimized for eukaryotic genomes. Bioinformatics 2014;30(8):1081–6. 24371153 10.1093/bioinformatics/btt727

[30] Rajan I, Aravamuthan S, Mande SS. Identification of compositionally distinct regions in genomes using the centroid method. Bioinformatics 2007;23:2672–7. 17724060 10.1093/bioinformatics/btm405

[31] Shrivastava S, Reddy CVSK, Mande SS. INDeGenIUS, a new method for high-throughput identification of specialized functional islands in completely sequenced organisms. J Biosci 2010;35:351–64. 20826944 10.1007/s12038-010-0040-4

[32] Vernikos GS, Parkhill J. Interpolated variable order motifs for identification of horizontally acquired DNA: revisiting the *Salmonella* pathogenicity islands. Bioinformatics 2006;22(18):2196–203. 16837528 10.1093/bioinformatics/btl369

[33] Chatterjee R, Chaudhuri K, Chaudhuri P. On detection and assessment of statistical significance of genomic islands. BMC Genomics 2008;9(1):150. 18380895 10.1186/1471-2164-9-150 PMC2362129

[34] de Brito DM, Maracaja-Coutinho V, de Farias ST, et al A novel method to predict genomic islands based on mean shift clustering algorithm. PLoS One 2016;11(1):e0146352. 26731657 10.1371/journal.pone.0146352 PMC4711805

[35] Arvey AJ, Azad RK, Raval A, et al Detection of genomic islands via segmental genome heterogeneity. Nucleic Acids Res 2009;37:5255–66. 19589805 10.1093/nar/gkp576 PMC2760805

[36] Zhang R, Ou H-Y, Gao F, et al Identification of horizontally-transferred genomic islands and genome segmentation points by using the GC profile method. Curr Genomics 2014;15:113–21. 24822029 10.2174/1389202915999140328163125 PMC4009839

[37] Wei W, Gao F, Du MZ, et al Zisland Explorer: detect genomic islands by combining homogeneity and heterogeneity properties. Brief Bioinform 2017;18:357–66. 26992782 10.1093/bib/bbw019 PMC5429010

[38] Langille MGI, Hsiao WWL, Brinkman FSL. Evaluation of genomic island predictors using a comparative genomics approach. BMC Bioinformatics 2008;9(1):329. 18680607 10.1186/1471-2105-9-329 PMC2518932

[39] Bertelli C, Brinkman FSL. Improved genomic island predictions with IslandPath-DIMOB. Bioinformatics 2018, in press. doi: 10.1093/bioinformatics/bty095. 10.1093/bioinformatics/bty095 PMC6022643 29905770

[40] Waack S, Keller O, Asper R, et al Score-based prediction of genomic islands in prokaryotic genomes using hidden Markov models. BMC Bioinformatics 2006;7:142. 16542435 10.1186/1471-2105-7-142 PMC1489950

[41] Vernikos GS, Parkhill J. Resolving the structural features of genomic islands: a machine learning approach. Genome Res 2008;18(2):331–42. 18071028 10.1101/gr.7004508 PMC2203631

[42] Che D, Hockenbury C, Marmelstein R, et al Classification of genomic islands using decision trees and their ensemble algorithms. BMC Genomics 2010;11(Suppl 2):S1. 10.1186/1471-2164-11-S2-S1 PMC2975412 21047376

[43] Han Wang DC, Wang H, Fazekas J, et al An accurate genomic island prediction method for sequenced bacterial and archaeal genomes. J Proteomics Bioinform 2014;7:214–21.

[44] Hudson CM, Lau BY, Williams KP. Islander: a database of precisely mapped genomic islands in tRNA and tmRNA genes. Nucleic Acids Res 2015;43:D48–53. 25378302 10.1093/nar/gku1072 PMC4383910

[45] Darling ACE, Mau B, Blattner FR, et al Mauve: multiple alignment of conserved genomic sequence with rearrangements. Genome Res 2004;14(7):1394–403. 15231754 10.1101/gr.2289704 PMC442156

[46] Altschul SF, Madden TL, Schäffer AA, et al Gapped BLAST and PSI-BLAST: a new generation of protein database search programs. Nucleic Acids Res 1997;25(17):3389–402. 9254694 10.1093/nar/25.17.3389 PMC146917

[47] Bertelli C, Laird MR, Williams KP, et al IslandViewer 4: expanded prediction of genomic islands for larger-scale datasets. Nucleic Acids Res 2017;45(W1):W30–5. 28472413 10.1093/nar/gkx343 PMC5570257

[48] Ou H-Y, Chen L-L, Lonnen J, et al A novel strategy for the identification of genomic islands by comparative analysis of the contents and contexts of tRNA sites in closely related bacteria. Nucleic Acids Res 2006;34(1):e3. 16414954 10.1093/nar/gnj005 PMC1326021

[49] Ou HY, He X, Harrison EM, et al MobilomeFINDER: web-based tools for in silico and experimental discovery of bacterial genomic islands. Nucleic Acids Res 2007;35:W97. 17537813 10.1093/nar/gkm380 PMC1933208

[50] Podell S, Gaasterland T. DarkHorse: a method for genome-wide prediction of horizontal gene transfer. Genome Biol 2007;8:R16. 17274820 10.1186/gb-2007-8-2-r16 PMC1852411

[51] Che D, Hasan MS, Wang H, et al EGID: an ensemble algorithm for improved genomic island detection in genomic sequences. Bioinformation 2011;7:311–14. 22355228 10.6026/007/97320630007311 PMC3280502

[52] Hasan MS, Liu Q, Wang H, et al GIST: genomic island suite of tools for predicting genomic islands in genomic sequences. Bioinformation 2012;8:203–5. 22419842 10.6026/97320630008203 PMC3302003

[53] Soares SC, Abreu VAC, Ramos RTJ, et al PIPS: pathogenicity island prediction software. PLoS One 2012;7(2):e30848. 22355329 10.1371/journal.pone.0030848 PMC3280268

[54] Soares SC, Geyik H, Ramos RTJ, et al GIPSy: genomic island prediction software. J Biotechnol 2016;232:2–11. 26376473 10.1016/j.jbiotec.2015.09.008

[55] Li J, Tai C, Deng Z, et al VRprofile: gene-cluster-detection-based profiling of virulence and antibiotic resistance traits encoded within genome sequences of pathogenic bacteria. Brief Bioinform 2017, in press. doi: 10.1093/bib/bbw141. 10.1093/bib/bbw141 28077405

[56] Jia B, Raphenya AR, Alcock B, et al CARD 2017: expansion and model-centric curation of the comprehensive antibiotic resistance database. Nucleic Acids Res 2017;45(D1):D566–73. 27789705 10.1093/nar/gkw1004 PMC5210516

[57] Dhillon BK, Chiu TA, Laird MR, et al IslandViewer update: improved genomic island discovery and visualization. Nucleic Acids Res 2013;41(W1):W129. 23677610 10.1093/nar/gkt394 PMC3692065

[58] Yoon SH, Park Y-K, Kim JF. PAIDB v2.0: exploration and analysis of pathogenicity and resistance islands. Nucleic Acids Res 2015;43:D624–30. 25336619 10.1093/nar/gku985 PMC4384037

[59] Bi D, Xu Z, Harrison EM, et al ICEberg: a web-based resource for integrative and conjugative elements found in Bacteria. Nucleic Acids Res 2012;40:D621–6. 22009673 10.1093/nar/gkr846 PMC3244999

[60] Podell S, Gaasterland T, Allen EE. A database of phylogenetically atypical genes in archaeal and bacterial genomes, identified using the DarkHorse algorithm. BMC Bioinformatics 2008;9(1):419. 18840280 10.1186/1471-2105-9-419 PMC2573894

[61] Dhillon BK, Laird MR, Shay JA, et al IslandViewer 3: more flexible, interactive genomic island discovery, visualization and analysis. Nucleic Acids Res 2015;43:W104–8. 25916842 10.1093/nar/gkv401 PMC4489224

[62] Tsirigos A, Rigoutsos I. A sensitive, support-vector-machine method for the detection of horizontal gene transfers in viral, archaeal and bacterial genomes. Nucleic Acids Res 2005;33(12):3699–707. 16006619 10.1093/nar/gki660 PMC1174904

[63] Tu Q, Ding D. Detecting pathogenicity islands and anomalous gene clusters by iterative discriminant analysis. FEMS Microbiol Lett 2003;221(2):269–75. 12725938 10.1016/S0378-1097(03)00204-0

[64] Lee CC, Chen YPP, Yao TJ, et al GI-POP: a combinational annotation and genomic island prediction pipeline for ongoing microbial genome projects. Gene 2013;518(1):114–23. 23318308 10.1016/j.gene.2012.11.063

[65] Assefa S, Keane TM, Otto TD, et al ABACAS: algorithm-based automatic contiguation of assembled sequences. Bioinformatics 2009;25(15):1968–9. 19497936 10.1093/bioinformatics/btp347 PMC2712343

[66] Pundhir S, Vijayvargiya H, Kumar A, PredictBias: a server for the identification of genomic and pathogenicity islands in prokaryotes. In Silico Biol 2008;8: 223–34. 19032158

[67] Langille MGI, Brinkman FSL. IslandViewer: an integrated interface for computational identification and visualization of genomic islands. Bioinformatics 2009;25(5):664–5. 19151094 10.1093/bioinformatics/btp030 PMC2647836

[68] Che D, Wang H. GIV: a tool for genomic islands visualization. Bioinformation 2013;9(17):879–82. 24250116 10.6026/97320630009879 PMC3819575

[69] Krzywinski M, Schein J, Birol I, et al Circos: an information aesthetic for comparative genomics. Genome Res 2009;19(9):1639–45. 19541911 10.1101/gr.092759.109 PMC2752132

[70] Yang J, Chen L, Sun L, et al VFDB 2008 release: an enhanced web-based resource for comparative pathogenomics. Nucleic Acids Res 2008;36:D539–42. 17984080 10.1093/nar/gkm951 PMC2238871

[71] Mao C, Abraham D, Wattam AR, et al Curation, integration and visualization of bacterial virulence factors in PATRIC. Bioinformatics 2015;31(2):252–8. 25273106 10.1093/bioinformatics/btu631 PMC4287947

[72] Laird MR, Langille MGI, Brinkman FSL. GenomeD3Plot: a library for rich, interactive visualizations of genomic data in web applications. Bioinformatics 2015;31:3348–9. 26093150 10.1093/bioinformatics/btv376 PMC4595901

[73] Bertelli C, Greub G. Rapid bacterial genome sequencing: methods and applications in clinical microbiology. Clin Microbiol Infect 2013;19:803–13. 23601179 10.1111/1469-0691.12217

[74] Fricke WF, Rasko DA. Bacterial genome sequencing in the clinic: bioinformatic challenges and solutions. Nat Rev Genet 2014;15(1):49–55. 24281148 10.1038/nrg3624

[75] Hingston P, Chen J, Dhillon BK, et al Genotypes associated with *Listeria monocytogenes* isolates displaying impaired or enhanced tolerances to cold, salt, acid, or desiccation stress. Front Microbiol 2017;8:369. 28337186 10.3389/fmicb.2017.00369 PMC5340757

[76] Winstanley C, Langille MGI, Fothergill JL, et al Newly introduced genomic prophage islands are critical determinants of *in vivo* competitiveness in the Liverpool epidemic strain of *Pseudomonas aeruginosa*. Genome Res 2008;19(1):12–23. 19047519 10.1101/gr.086082.108 PMC2612960

[77] Ingle DJ, Tauschek M, Edwards DJ, et al Evolution of atypical enteropathogenic *E. coli* by repeated acquisition of LEE pathogenicity island variants. Nat Microbiol 2016;1(2):15010. 27571974 10.1038/nmicrobiol.2015.10

[78] Mantri Y, Williams KP Islander: a database of integrative islands in prokaryotic genomes, the associated integrases and their DNA site specificities. Nucleic Acids Res 2004;32:55D–58. 10.1093/nar/gkh059 PMC308793 14681358

[79] Metzler S, Kalinina OV Detection of atypical genes in virus families using a one-class SVM. BMC Genet 2014;15:913. 10.1186/1471-2164-15-913 PMC4210486 25336138

[80] Elhai J, Liu H, Taton A Detection of horizontal transfer of individual genes by anomalous oligomer frequencies. BMC Genet 2012;13:245. 10.1186/1471-2164-13-245 PMC3497702 22702893

[81] Jain R, Ramineni S, Parekh N IGIPT - Integrated genomic island prediction tool. Bioinformation 2011;7:307–10. 22355227 10.6026/007/97320630007307 PMC3280501

